# Lack of miRNA Misregulation at Early Pathological Stages in *Drosophila* Neurodegenerative Disease Models

**DOI:** 10.3389/fgene.2012.00226

**Published:** 2012-10-30

**Authors:** Anita Reinhardt, Sébastien Feuillette, Marlène Cassar, Céline Callens, Hélène Thomassin, Serge Birman, Magalie Lecourtois, Christophe Antoniewski, Hervé Tricoire

**Affiliations:** ^1^Laboratoire de Génétique du Stress et du Vieillissement, Unité de Biologie Fonctionnelle et Adaptative, CNRS EAC 4413, Université Paris DiderotSorbonne Paris Cité, Paris, France; ^2^INSERM, U1079Rouen, France; ^3^Institute for Research and Innovation in Biomedicine, University of RouenRouen, France; ^4^Genetics and Physiopathology of Neurotransmission, Neurobiology Unit, CNRSESPCI ParisTech, Paris, France; ^5^Génétique et Epigénétique de la Drosophile, UMR 7622 Biologie du Développement, UPMC-CNRS 9Paris, France

**Keywords:** miRNA, neurodegenerative diseases, ataxia, frontotemporal lobar degeneration, Parkinson disease, polyQ diseases, deep sequencing

## Abstract

Late onset neurodegenerative diseases represent a major public health concern as the population in many countries ages. Both frequent diseases such as Alzheimer disease (AD, 14% incidence for 80–84 year-old Europeans) or Parkinson disease (PD, 1.4% prevalence for >55 years old) share, with other low-incidence neurodegenerative pathologies such as spinocerebellar ataxias (SCAs, 0.01% prevalence) and frontotemporal lobar degeneration (FTLD, 0.02% prevalence), a lack of efficient treatment in spite of important research efforts. Besides significant progress, studies with animal models have revealed unexpected complexities in the degenerative process, emphasizing a need to better understand the underlying pathological mechanisms. Recently, microRNAs (miRNAs), a class of small regulatory non-coding RNAs, have been implicated in some neurodegenerative diseases. The current data supporting a role of miRNAs in PD, tauopathies, dominant ataxias, and FTLD will first be discussed to emphasize the different levels of the pathological processes which may be affected by miRNAs. To investigate a potential involvement of miRNA dysregulation in the early stages of these neurodegenerative diseases we have used *Drosophila* models for seven diseases (PD, 3 FTLD, 3 dominant ataxias) that recapitulate many features of the human diseases. We performed deep sequencing of head small RNAs after 3 days of pathological protein expression in the fly head neurons. We found no evidence for a statistically significant difference in miRNA expression in this early stage of the pathological process. In addition, we could not identify small non-coding CAG repeat RNAs (sCAG) in polyQ disease models. Thus our data suggest that transcriptional deregulation of miRNAs or sCAG is unlikely to play a significant role in the initial stages of neurodegenerative diseases.

## Introduction

Late onset human neurodegenerative diseases are characterized by neuronal dysfunction, progressive degeneration, and progressive tissue specific neuronal loss. They include common diseases such as Alzheimer disease (AD, 14% incidence for 80–84 year-old Europeans) or Parkinson disease (PD, 1.4% prevalence for >55 years old), but also a large number of other pathologies presenting lower incidences such as spinocerebellar ataxias (SCAs, 0.01% prevalence) and frontotemporal lobar degeneration (FTLD, 0.02% prevalence). In spite of the large clinical heterogeneity of these diseases, age is the most prominent risk factor for all of them (Yankner et al., 2008; Brookmeyer et al., 2011). Consequently, since the mean age of world population is steadily increasing, they are becoming a major health concern and economic burden in both developed and developing countries.

Although, for some of the diseases, sporadic cases are the most frequent, investigations conducted during the last decades have identified key genes associated with familial forms. This paved the way to myriads of studies using either cells or animal models to understand the physiological basis of these diseases, with significant success. A common hallmark of several of these aging-associated neurodegenerative disorders that emerged from these early studies, is the existence of fibrillar aggregates of aggregation-prone proteins that may trap other cellular compounds (Jucker and Walker, 2011). Impairment of protein quality control homeostasis during aging or in response to environmental or endogenous stresses (notably oxidative stress) may thus contribute to neuronal dysfunction. However, whether such aggregates or intermediate forms are toxic or protective is still a matter of debate. Additionally, animal models have revealed unexpected complexities in the degenerative process that cannot be reduced to a single pathological mechanism. Thus, much remains to be learned of the mechanisms leading to specific neuronal death in late onset diseases.

MicroRNAs (miRNAs) have been recently identified as potential important players in neurodegenerative diseases. miRNAs are small non-coding RNAs, present in many multicellular organisms including humans, that post-transcriptionally regulate the expression of their target genes by inhibition of mRNA translation or mRNA degradation (Krol et al., 2010). Since miRNAs can simultaneously and rapidly target hundreds of genes, it is not surprising that they have been shown to play an important role in many developmental processes and in cellular homeostasis (Kloosterman and Plasterk, 2006; Bushati and Cohen, 2007). Due to these crucial functions, their activity has to be tightly regulated at many levels.

Besides minor alternative pathways, the canonical biogenesis pathway of miRNAs involves (1) the synthesis of a long primary transcript (pri-miRNA), (2) the cleavage of the pri-miRNA into a 70–100 nt precursor (pre-miRNA) inside a nuclear multiprotein complex called the Microprocessor containing the RNase III Drosha, (3) the transport of the pre-miRNA into the cytoplasm where it is cleaved by another RNase III enzyme, Dicer, into a 21–22 nt long duplex, and (4) the association of one the strands of the duplex (the active miRNA) with an Argonaute (Ago) protein within the RISC complex, where it plays its repressive role. Due to this complex process, the level of expression of a miRNA may be regulated at several levels. One standard way is through transcriptional regulation of the pri-miRNA synthesis that will impact the level of miRNA. This accounts for a large part of the complex temporal and spatial regulation of the miRNAs inside the central nervous system (Kosik, 2006; Kapsimali et al., 2007; Bak et al., 2008). However, many post-transcriptional mechanisms of miRNA regulation have also been described (reviewed in; Newman and Hammond, 2010; Siomi and Siomi, 2010). They involve either cofactors present inside the Microprocessor complex (Gregory et al., 2004) or RNA-binding proteins that may regulate the processing of specific subsets of pri-miRNAs, the positive or negative regulation of pre-miRNA processing by Dicer, and the control of miRNA turnover.

MicroRNAs have been implicated in neurodegenerative diseases in several ways and can be classified accordingly (reviewed in; Bushati and Cohen, 2007; Eacker et al., 2009; Gascon and Gao, 2012; Kaur et al., 2012). First, it has been shown in several organisms and in various neuronal subpopulations that disruption of the miRNA pathway through Dicer depletion impacts neuronal differentiation and survival (Schaefer et al., 2007; Cuellar et al., 2008). Similarly, targeted depletion of Dicer in astrocytes, oligodendrocytes, and Schwann cells leads to neurodegeneration (Shin et al., 2009; Pereira et al., 2010; Tao et al., 2011), pointing out a potential role of glial miRNAs in neurodegenerative diseases. Interestingly, lack of Atxn2, the protein involved in SCA2 disease which interacts with Ago1, impairs the repressive activity of several miRNAs (McCann et al., 2011). However, it is not known whether this dysfunction is physiologically relevant to the SCA2 pathology.

Second, several miRNAs (referred to below as class I neurodegenerative disease-associated miRNAs; NDAmiR) have been shown to target some disease-related proteins and modulate their cellular concentration. For instance, in AD, several miRNAs are able to modulate Aβ peptide production through APP or BACE1 targeting, or repress genes implicated in the phosphorylation state of the Tau protein (reviewed in; Delay et al., 2012). Similarly, several miRNAs may target α-synuclein, a protein linked to PD, while Ataxin-1, the protein implicated in SCA1 disease, is regulated by at least 4 miRNAs (Gascon and Gao, 2012; and references therein). However these studies have been conducted *in vitro* and it is not currently known whether these regulations are physiologically important in the course of these diseases.

Finally, recent data suggest that miRNA dysregulation may represent an important part of pathological mechanisms involved in neurodegenerative diseases. Dysregulated miRNAs (referred to below as class II NDAmiR) have several origins. Two proteins mutated in familial cases of amyotrophic lateral sclerosis (ALS) or FTLD, the RNA-binding proteins TAR DNA-binding protein-43 (TDP-43) and fused in sarcoma (FUS), have been identified in Microprocessor complexes (Gregory et al., 2004; Kawahara and Mieda-Sato, 2012). Furthermore, TDP-43 also interacts with the RISC complex and is required for the correct expression of a subset of miRNAs in cell cultures (Kawahara and Mieda-Sato, 2012). Therefore, some disease-related proteins may directly dysregulate the expression of some miRNAs (class IIa) through their biogenesis pathway. In contrast to these direct evidences, aberrant expression of miRNAs (class IIb) have been found in a variety of animal models of neurodegenerative diseases and in post-mortem brain samples of AD, PD, and Huntington disease (HD) patients (reviewed in; Lau and de Strooper, 2010; Gascon and Gao, 2012). In only a few cases potential dysregulation mechanisms could be proposed. For instance, in the case of HD, inhibition of the REST co-repressor by the pathological Htt protein likely results in overexpression of at least four neuronal miRNAs (Johnson et al., 2008; Packer et al., 2008). However, several caveats may complicate the interpretation of these data. First, technical issues, such as the stability of miRNAs during the analysis of brains from patients may have led to false positive detection (Sethi and Lukiw, 2009). Then, most studies focused primarily on neuron dysfunctions, and the relative contribution of neurons and glial cells in miRNA dysregulation *in vivo* was not addressed. However, glial inflammatory responses are observed in many of these diseases and could be responsible for a significant part of miRNA transcriptome modifications. Finally, analyses were usually performed at advanced stages of the diseases where neuronal loss is frequently observed. The observed differences in miRNA concentration may therefore arise from differences in tissue composition compared to the control samples. Alternatively, they may represent unspecific events consecutive to secondary processes occurring in neurodegeneration such as protein homeostasis perturbations or generation of oxidative stress. A critical issue is thus to decipher whether miRNA dysregulation can be observed at the beginning of the pathological process and, subsequently, play a significant role in the evolution of the disease.

In this paper we addressed this issue in *Drosophila* models related to seven different neurodegenerative diseases (PD, 3 FTLD, 3 dominant ataxias). These models were previously shown to recapitulate many features of human diseases and are amenable to subsequent genetic analysis of miRNAs of interest. We used genetic tools to express pathological proteins specifically in neurons and performed a miRNA profiling by deep sequencing. We found no evidence for a statistically significant difference in miRNA expression in this early stage of the pathological process. Thus our data suggest that transcriptional deregulation of miRNAs is unlikely to play a significant role in the initial stages of neurodegenerative diseases.

## Materials and Methods

### *Drosophila* strains and husbandry

For SCA models, the following lines were used: UAS-Atxn1-82Q and UAS-Atxn1-30Q (Fernandez-Funez et al., 2000), UAS-Atxn3-70Q and UAS-Atxn3-19Q, UAS-Atxn7-102Q and UAS-Atxn7-10Q (Latouche et al., 2007). The Atxn3 lines were generated by cloning of the full length Atxn3 coding region described in (Mueller et al., 2009) inside a pAttB-UAS vector followed by insertion at the Att40 landing site by standard ways. For FTLD diseases the following transgenic *Drosophila* strains were used in this study: UAS-TauV337M (Wittmann et al., 2001); UAS-TDP-43 (Miguel et al., 2011); and UAS-FUS (Miguel et al., 2012). *Drosophila* strains were raised on standard cornmeal-yeast agar medium. Fly cultures and crosses were carried out at 25°C.

Males from these strains were crossed with elav-GAL4-GeneSwitch (ElavGal4^GS^) females (Osterwalder et al., 2001). Depending on the protein of interest, more than 500 males or females (0–2 days old) of the progeny were collected (clusters of 25–30) into food tubes containing standard cornmeal-yeast agar medium. After 7 days, the flies were transferred to new tubes containing instant *Drosophila* medium (Carolina Biological Supply Company, Burlington, NC, USA) or corn yeast medium (SCAs experiments) with or without RU486 (Mifepristone, Betapharma-Shanghai Co., Ltd., China) at a final concentration of 1% ethanol and 200 μg/ml RU486. Two days later, the flies were transferred to new tubes containing fresh media for one more day. After these 3 days of RU486 induction, flies were quickly frozen in liquid nitrogen and then stores at −80°C.

For the PD experiments, the following *Drosophila* strains were used: Canton S, *w*^1118^, *elav-GAL4*, *UAS-*α*-synuclein*, and *UAS-*α*-synuclein-A30P* (Feany and Bender, 2000). For α*-synuclein* expression in neurons, *elav-GAL4* virgins were crossed to *UAS-*α*-synuclein* or *UAS-*α*-synuclein-A30P* males, or to *w*^1118^ for controls. 7 to 10-day-old non-virgin females of the F1 progeny were collected (300–350 flies per genotype) and kept at −80°C until RNA extraction.

Paraquat treatment was performed on 7 to 10-day-old Canton S adult females by dietary ingestion. Fifty flies were incubated at 25°C in a 8.5 cm diameter Petri dish containing two layers of Whatman paper soaked with 20 mM paraquat (methyl viologen, Sigma-Aldrich, St Louis, MO, USA) diluted in 2% (wt/vol) sucrose or sucrose only for controls. Fourteen similar Petri dishes were independently treated for each condition. After 24 h, around 300 surviving flies were collected and frozen at −80°C.

### Total RNA isolation

For each head sample, 300 adult fly were freezed in liquid nitrogen and the heads were recovered by sieving. For each body sample used in control experiment, 50 flies were freezed in liquid nitrogen. These frozen samples were directly transferred into a Lysing Matrix D tube containing Lysing Matrix beads (Qbiogen) and 1 ml of qiazol (Qiagen) and grinded with a FastPrep Homogenizer (MP Biomedicals). The homogenate was incubated at room temperature for 5 min and then centrifuged at 12,000 *g* for 10 min at 4°C to remove cellular debris. The supernatant was transferred to a fresh tube, and 200 μl of chloroform were added per ml of Qiazol. After vortex agitation for 30 s, samples were incubated at room temperature for 3 min and then centrifuged at 16,000 *g* for 15 min at 4°C. The aqueous phase was transferred to a fresh tube, and RNA was precipitated by adding 1 volume of isopropanol. Samples were incubated for 1 h at −20°C and then centrifuged at 12,000 *g* for 30 min at 4°C. The RNA pellet was washed by adding 600 μl of cold 80% ethanol and centrifuged at 16,000 *g* for 10 min at 4°C. The ethanol was then discarded and the pellet air dried for 10 min.

The RNA pellet was dissolved in of Rnase-free water and 250 μl of acid phenol-chlorofom-IAA 125:24:1 (pH 4.5; Ambion) were added. After vortex agitation for 30 s, samples were centrifuged at 16,000 *g* for 10 min at 4°C. The aqueous phase was transferred to a fresh tube, and 250 μl of chloroform were added. After vortex agitation for 15 s, samples were centrifuged at 16,000 *g* for 10 min at 4°C. The aqueous phase was transferred to a fresh tube, and 1/10 volume of NaAc (3M, pH 5.0) and 4 volumes of cold 100% ethanol were added. Samples were then incubated overnight at −20°C for RNAs precipitation and centrifuged at 16,000 g for 30 min at 4°C. The RNA pellet was washed with 600 μl of cold 80% ethanol and centrifuged at 16,000 *g* for 10 min at 4°C. After a second wash step and centrifugation, the RNA pellet was air dried for 10 min and in 50 μl of RNA-free water. Before being further processed, the concentration of RNA samples was measured by spectrophotometry and their quality was checked with an Agilent 2100 Bioanalyzer (Agilent Technologies, Palo Alto, USA).

### Deep sequencing and data analysis

Small RNAs from fly head RNAs were cloned using the using the TruSeq (TM) SBS v5 Kit and sequenced using an Illumina Hi-Seq 2000 at Fasteris (http://www.fasteris.com/). Sequence reads in fastq format were trimmed from the adapter sequence 5′-CTGTAGGCACCATCAAT-3′ and reads with more than 18 nt were matched to the *Drosophila melanogaster* genome release 5.43 using Bowtie and allowing 0 or 1 mismatch (-v1 option). Matched reads were then re-matched against (1) the miRBase r18 miRNA stem-loop sequences. Reads matching these sequences with 0 or 1 mismatch were retained for subsequent analysis whereas unmatched reads were re-matched to (2) other non-coding RNA sequences (tRNAs, rRNAs, and miscellaneous ncRNAs). Reads matching these sequences with 0 or 1 mismatch were counted whereas unmatched reads were re-matched to (3) transposon element sequences. This procedure was further iteratively applied to (4) introns, (5) mRNAs, and (6) intergenic sequences to produce the annotations in Table 1.

**Table 1:** **miRNAs implicated in neurodegenerative diseases belonging to conserved miRNA families with *Drosophila* members**.

Identified miR	Family_ID	Family_name	dme-miR	Disease	Reference	Variation
hsa-let-7b	MIPF0000002	let-7	dme-let-7	AD	Wang et al. (2011)	Down
hsa-let-7c	MIPF0000002	let-7	dme-let-7	AD	Wang et al. (2011)	Down
hsa-let-7e	MIPF0000002	let-7	dme-let-7	AD	Wang et al. (2011)	Up
hsa-let-7g	MIPF0000002	let-7	dme-let-7	AD	Schonrock et al. (2010)	Down
hsa-let-7g	MIPF0000002	let-7	dme-let-7	AD	Wang et al. (2011)	Down
hsa-let-7i	MIPF0000002	let-7	dme-let-7	AD	Schonrock et al. (2010)	Down
hsa-let-7i	MIPF0000002	let-7	dme-let-7	AD	Wang et al. (2011)	Down
hsa-miR-98	MIPF0000002	let-7	dme-let-7	AD	Wang et al. (2011)	Down
mmu-let-7b	MIPF0000002	let-7	dme-let-7	AD	Wang et al. (2009)	Up
mmu-let-7d	MIPF0000002	let-7	dme-let-7	AD	Wang et al. (2009)	Up
mmu-let-7e	MIPF0000002	let-7	dme-let-7	AD	Wang et al. (2009)	Up
mmu-mir-98	MIPF0000002	let-7	dme-let-7	AD	Wang et al. (2009)	Up
cel-let-7	MIPF0000002	let-7	dme-let-7	PD	Asikainen et al. (2010)	Down
dme-let-7	MIPF0000002	let-7	dme-let-7	PD	Gehrke et al. (2010)	NC
hsa-let-7c	MIPF0000002	let-7	dme-let-7	PolyQ-HD	Martí et al. (2010)	Down
hsa-let-7d	MIPF0000002	let-7	dme-let-7	PolyQ-HD	Martí et al. (2010)	Down
hsa-let-7e	MIPF0000002	let-7	dme-let-7	PolyQ-HD	Martí et al. (2010)	Down
hsa-let-7g	MIPF0000002	let-7	dme-let-7	PolyQ-HD	Martí et al. (2010)	Up
hsa-let-7i	MIPF0000002	let-7	dme-let-7	PolyQ-HD	Martí et al. (2010)	Up
hsa-miR-98	MIPF0000002	let-7	dme-let-7	PolyQ-HD	Martí et al. (2010)	Down
hsa-miR-98	MIPF0000002	let-7	dme-let-7	PolyQ-HD	Martí et al. (2010)	Up
cel-miR-1	MIPF0000038	mir-1	dme-mir-1	PD	Asikainen et al. (2010)	Down
hsa-miR-99a	MIPF0000025	mir-99	dme-mir-100	AD	Wang et al. (2011)	Down
hsa-miR-99b	MIPF0000025	mir-99	dme-mir-100	AD	Wang et al. (2011)	Down
hsa-miR-100	MIPF0000025	mir-99	dme-mir-100	PolyQ-HD	Martí et al. (2010)	Up
hsa-miR-99a	MIPF0000025	mir-99	dme-mir-100	PolyQ-HD	Martí et al. (2010)	Up
hsa-miR-99b	MIPF0000025	mir-99	dme-mir-100	PolyQ-HD	Martí et al. (2010)	Up
mmu-mir-125a	MIPF0000017	mir-125	dme-mir-125	AD	Wang et al. (2009)	Up
hsa-miR-133b	MIPF0000029	mir-133	dme-mir-133	PD	Kim et al. (2007)	Down
hsa-miR-137	MIPF0000106	mir-137	dme-mir-137	AD	Geekiyanage and Chan (2011)	Down
mmu-miR-137	MIPF0000106	mir-137	dme-mir-137	AD	Schonrock et al. (2010)	Down
hsa-miR-137	MIPF0000106	mir-137	dme-mir-137	PolyQ-HD	Martí et al. (2010)	Down
hsa-mir-137	MIPF0000106	mir-137	dme-mir-137	Tauopathies	Smith et al. (2011)	NC
hsa-miR-184	MIPF0000059	mir-184	dme-mir-184	AD	Wang et al. (2011)	Up
hsa-miR-184	MIPF0000059	mir-184	dme-mir-184	PolyQ-HD	Martí et al. (2010)	Down
hsa-miR-193b	MIPF0000082	mir-193	dme-mir-193	AD	Wang et al. (2011)	Down
hsa-miR-193b	MIPF0000082	mir-193	dme-mir-193	PolyQ-HD	Martí et al. (2010)	Up
hsa-miR-210	MIPF0000086	mir-210	dme-mir-210	AD	Hebert et al. (2008)	Down
hsa-miR-210	MIPF0000086	mir-210	dme-mir-210	PolyQ-HD	Martí et al. (2010)	Up
hsa-mir-29a	MIPF0000009	mir-29	dme-mir-285	AD	Geekiyanage and Chan (2011)	Down
hsa-miR-29a	MIPF0000009	mir-29	dme-mir-285	AD	Shioya et al. (2010)	Down
hsa-miR-29a	MIPF0000009	mir-29	dme-mir-285	AD	Wang et al. (2011)	Down
hsa-miR-29b-1	MIPF0000009	mir-29	dme-mir-285	AD	Hebert et al. (2008)	Down
hsa-miR-29c	MIPF0000009	mir-29	dme-mir-285	AD	Wang et al. (2011)	Down
mmu-mir-29a	MIPF0000009	mir-29	dme-mir-285	AD	Wang et al. (2009)	Down
mmu-mir-29c	MIPF0000009	mir-29	dme-mir-285	AD	Wang et al. (2009)	Down
hsa-mir-29a	MIPF0000009	mir-29	dme-mir-285	PolyQ-HD	Johnson et al. (2008)	Up
hsa-miR-29c	MIPF0000009	mir-29	dme-mir-285	PolyQ-HD	Martí et al. (2010)	Down
hsa-miR-29c	MIPF0000009	mir-29	dme-mir-285	PolyQ-HD	Martí et al. (2010)	Up
mmu-mir-29a	MIPF0000009	mir-29	dme-mir-285	PolyQ-HD	Johnson et al. (2008)	Down
mmu-miR-29c	MIPF0000009	mir-29	dme-mir-285	PolyQ-HD	Lee et al. (2008)	Down
hsa-miR-33a	MIPF0000070	mir-33	dme-mir-33	AD	Wang et al. (2011)	Down
hsa-miR-33a	MIPF0000070	mir-33	dme-mir-33	PolyQ-HD	Martí et al. (2010)	Up
hsa-miR-33b	MIPF0000070	mir-33	dme-mir-33	PolyQ-HD	Martí et al. (2010)	Up
hsa-miR-34a	MIPF0000039	mir-34	dme-mir-34	AD	Lukiw and Alexandrov (2012)	Up
hsa-miR-34a	MIPF0000039	mir-34	dme-mir-34	AD	Wang et al. (2011)	Down
hsa-miR-34b	MIPF0000039	mir-34	dme-mir-34	AD	Wang et al. (2011)	Down
mmu-mir-34a	MIPF0000039	mir-34	dme-mir-34	AD	Wang et al. (2009)	Up
hsa-miR-34b	MIPF0000039	mir-34	dme-mir-34	PD	Minones-Moyano et al. (2011)	Down
hsa-miR-34c	MIPF0000039	mir-34	dme-mir-34	PD	Minones-Moyano et al. (2011)	Down
hsa-miR-375	MIPF0000114	mir-375	dme-mir-375	PolyQ-HD	Martí et al. (2010)	Down
hsa-miR-25	MIPF0000013	mir-25	dme-mir-92a	AD	Lukiw and Alexandrov (2012)	Up
hsa-miR-92b	MIPF0000013	mir-25	dme-mir-92a	AD	Wang et al. (2011)	Down
mmu-mir-92b	MIPF0000013	mir-25	dme-mir-92a	AD	Wang et al. (2009)	Up
hsa-miR-25	MIPF0000013	mir-25	dme-mir-92a	PolyQ-HD	Martí et al. (2010)	Up
hsa-miR-25	MIPF0000013	mir-25	dme-mir-92b	AD	Lukiw and Alexandrov (2012)	Up
hsa-miR-92b	MIPF0000013	mir-25	dme-mir-92b	AD	Wang et al. (2011)	Down
mmu-mir-92b	MIPF0000013	mir-25	dme-mir-92b	AD	Wang et al. (2009)	Up
hsa-miR-25	MIPF0000013	mir-25	dme-mir-92b	PolyQ-HD	Martí et al. (2010)	Up

Using the *miRNA_bowtie_profiler* python script (available upon request), we parsed the bowtie output files to count and map the miRNA reads to the miRNA stem-loop sequences. To assign read counts to either the 5p or 3p miRNAs, each miRNA stem-loop sequence was iteratively split between −20 and +20 nucleotides relative to the middle of the miRNA stem-loop sequence. For each split position, the numbers of reads mapping entirely to the upstream and downstream substrings were computed and stored, whereas reads mapping across the split position were discarded. Then, the split position for which the sum of the upstream and downstream reads was the nearest of the total number of reads mapped to the miRNA stem-loop sequence was retained, and the upstream and downstream read counts were assigned to the 5p and 3p miRNAs, respectively. Using this algorithm, miRNA5p and miRNA3p read counts were unambiguously assigned, independently of miRBase annotations for miRNA and miRNA* species. Note that in the case of non-canonical miRNAs with reads tilled across the stem-loop precursor, the procedure leads to arbitrarily assign read counts to miRNA5p and miRNA3p species which may not be biologically relevant; however, these counts still reflect the expression level of the miRNA stem-loop precursor.

Expression profiling of mature miRNAs was performed using the hit tables generated as described above and the DEseq R package (Anders and Huber, 2010). The recently improved version (1.7.6) was used, with the default options (notably sharing Mode = “maximum” in the estimate Dispersions function).

### Western blotting

Fifty adult fly heads were dissected and homogenized in 100 μl RIPA buffer [50 mM Tris-HCl, pH 8, 150 mM NaCl, 20 mM EDTA, 1% Nonidet-P40 (v/v), 50 mM natrium fluoride, 20 mM *N*-ethylmaleimide, and a cocktail of protease inhibitors from Sigma-Aldrich, St. Louis, MO, USA]. Samples were placed under agitation at 4°C for 1 h and then centrifuged at 11,300 *g* for 20 min at 4°C to remove cellular debris. Protein concentrations in RIPA fraction were measured using the DC Protein Assay Kit (Bio-Rad Laboratories, Hercules, CA, USA). Protein samples were loaded in SDS-PAGE sample buffer (240 mM Tris-HCl, pH 6.8, 6% SDS, 30% glycerol, 0.06% bromophenol blue) and resolved by a 10% SDS-PAGE. Proteins were transferred to nitrocellulose membranes (Hybond C-Extra; Amersham Biosciences, Arlington Heights, IL, USA). The following antibodies were used in this study: rabbit polyclonal anti-TARDBP/TDP-43 (1:5,000; Proteintech Group, Inc., Chicago, IL, USA) and rat monoclonal anti-elav (1:100, Developmental Studies Hybridoma Bank, Iowa City, IA, USA). Membranes were incubated with peroxidase-labeled anti-rat or anti-rabbit antibodies (1:10,000) from Jackson Immunoresearch Laboratories (WestGrove, PA, USA), and signals were detected with chemiluminescence reagents (GE Healthcare, Saclay, France). Signals were acquired with a GBOX (Syngene, Cambridge, UK) monitored by the Gene Snap software.

### Real-time quantitative RT–PCR

For each genotype, 1 μg of total RNA were first treated with Deoxyribonuclease I Amplification Grade (Sigma-Aldrich) and then reverse-transcribed into cDNA, using the First Strand cDNA Synthesis Kit (Amersham Biosciences). PCR reactions were performed in a final volume of 20 μl, using the SsoFast Evagreen Supermix (Bio-Rad, Hercules, CA, USA) with primers at a final concentration of 300 nM (primer sequences available upon request). PCR amplifications were performed on a CFX96 Real-Time System (Bio-Rad) using the following cycling steps: enzyme activation at 98°C for 2 min; denaturation and annealing/extension respectively at 98°C for 10 s and 60°C for 15 s (40 cycles). The comparative −ΔΔCt method was then used to determine quantitative values for gene expression levels in each sample using 14.3.3ε and *Cyp1* as normalizer genes.

## Results

### Generation of miRNA profiles at early disease stages in *Drosophila* models

**Figure 1:**
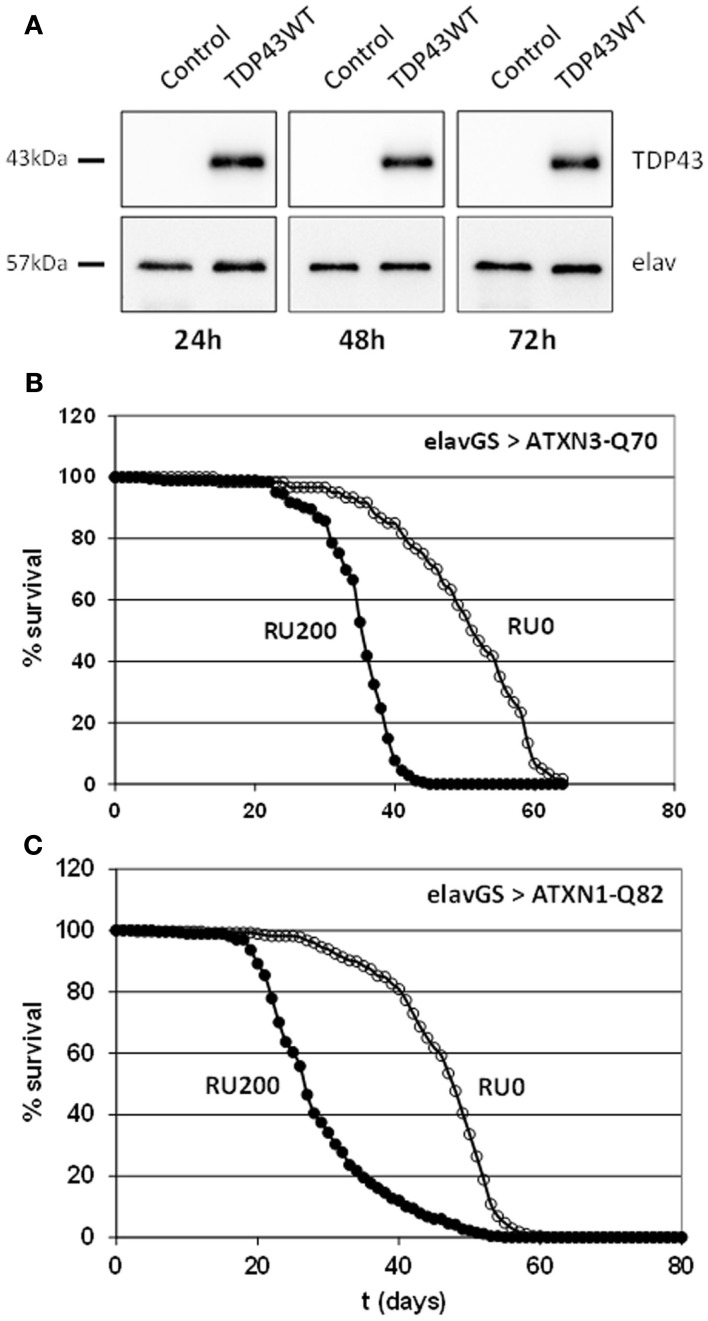
**(A)** Fast induced expression of TDP-43 in adult *Drosophila* neurons. Western blot analyses on total protein extracts prepared from heads of ElavGal4^GS^ flies in which expression of the human transgene was induced with 200 ng/μl of RU486 for 1, 2, or 3 days. Control flies: ElavGal4^GS^/+. elav is used as a loading control. TDP-43 proteins are readily detected 24 h after induction and the level of expression remains stable. **(B)** Toxicity of full length expanded ATXN3. Longevity of ElavGal4^GS^/UAS-ATXN3-Q70 flies was measured in conditions of neuronal protein induction (200 μg/ml of RU486 into food medium) or without induction (no RU486). Mean lifespan is significantly reduced from 51 to 35.3 days (69%) when the expanded protein is expressed. As already shown, expression of an unexpanded ATXN3-Q19 protein does not modify the lifespan. **(C)** Toxicity of full length expanded ATXN1. Longevity of ElavGal4^GS^/UAS-ATXN1-Q82 flies was measured in conditions of neuronal protein induction (200 μg/ml of RU486 into food medium) or without induction (no RU486). Mean lifespan is significantly reduced from 45 to 28 days (62%) when the expanded protein is expressed.

Among the miRNAs that have been proposed to be involved in neurodegenerative diseases in mammals, 30 of them belongs to a conserved miRNA family that has at least one member in *Drosophila* (Table 1). Surprisingly, we noticed in many cases opposite directions of transcriptional changes inside a given family and a given pathology. This points out the need of additional studies to uncover the functional significance of these observations, and, notably, to investigate whether miRNA dysregulation can be observed at the first stages of the diseases. Therefore our major aim was to focus on class II NDAmiR in *Drosophila* models of human neurodegenerative diseases that have been well characterized previously and amenable to early stage study. Thus we used dominant models of ataxias (SCA1, SCA3, SCA7) and FTLD (TDP-43, FUS, TAU) where pathological or control proteins can be targeted to neurons in a temporally controlled manner with the RU486 inducible ElavGal4^GS^ line (see below for complete genotype description). Importantly, since its first introduction in 2007 by one of us to modelize successfully SCA7 pathology (Latouche et al., 2007), the elavGS system has been used by several labs to study *Drosophila* neurodegenerative disease models. This includes a model for Aβ induced pathology (Sofola et al., 2010; Rogers et al., 2012), models for polyQ diseases (Spinobulbar muscular atrophy (SBMA; Pandey et al., 2007), and SCA3 (Martin-Lannerée et al., 2006), model for Parkinson disease (Kanao et al., 2010) as well as the two FTD models used in this study (FUS and TBP43; Lanson et al., 2011; Miguel et al., 2011, 2012). Therefore, it is now widely accepted that inducible elavGS models are adequate to express disease proteins in adult CNS with pathological consequences that mimic the disease. Flies were induced when 7 day-old, by incorporation of RU486 into their food, and RNA extraction was performed on 10 day-old fly heads. As expected from previous characterization of the GeneSwitch system (Osterwalder et al., 2001), proteins are readily detected 24 h after induction (Figure 1A). Thus, with this strategy, we are able to detect early transcriptome changes occurring during the first 3 days of the pathological process.

In addition to these dominant models, we used a model of sporadic PD where flies were treated during 2 days with the neurotoxic compound paraquat before RNA extraction on fly heads. We completed this study with another model of PD linked to α-synuclein overexpression. In all these cases the age of the flies selected for RNA extraction was 7 days. Therefore they were analyzed separately from the previous batch of samples.

**Figure 2:**
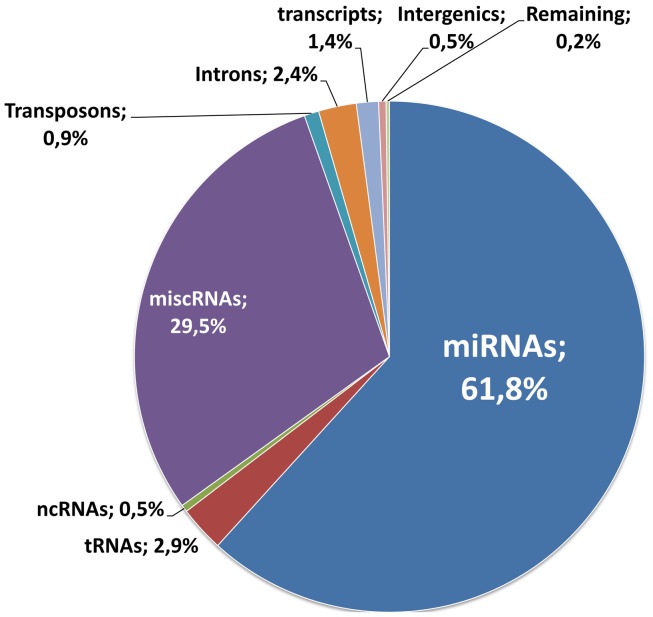
**Distribution of RNA reads**. 266 million reads were generated for the 36 samples of this study. They were matched to the different mutually exclusive categories, according to the procedure described in the Section “Material and Methods”: miRNAs, tRNAs, ncRNAs, miscRNAs, transposons, Introns, transcripts, and intergenic sequences. The distribution of the reads for each class is indicated on the graph.

**Figure 3:**
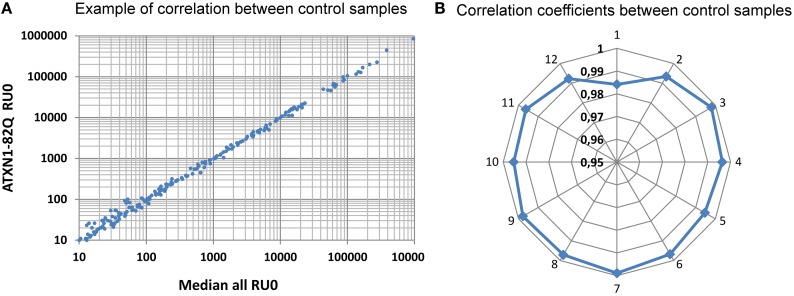
**Correlation between normalized reads**. Distribution of miRNAs read counts was compared between the median distribution of all the control samples and the different distributions. A typical correlation distribution is presented in **(A)** for the ATXN1-82Q RU0 sample. In **(B)** the distribution of the correlation coefficients for curves similar to **(A)** are displayed for all the ATXN samples of our first sequencing run. Notice that all except one values range above 0.99, reflecting the high correlation between samples. Sample identification: 1: ATXN3-70Q-0, 2: ATXN3-70Q-200, 3: ATXN3-19Q-0, 4: ATXN3-19Q-200, 5: ATXN1-82Q-0, 6: ATXN1-82Q-200, 7: ATXN1-30Q-0, 8: ATXN1-30Q-200, 9: ATXN7-102Q-0, 10: ATXN7-102Q-200, 11: ATXN7-10Q-0, 12: ATXN7-10Q-200.

Following RNA extraction and quality control, generation, and sequencing of small RNA libraries were performed on Illumina Hi-Seq. A total of 266 million reads were generated for the 36 samples of this study and were matched to different mutually exclusive categories (miRNAs, tRNAs, ncRNAs, miscRNAs, transposons, Introns, transcripts, and intergenic sequences). A detailed description for each sample is given in Table S1 in Supplementary Material. As a whole, as expected, the miRNAs class is the most abundant (61% of the total; Figure 2). With these selected reads, normalization between samples and subsequent statistical analysis were performed with Deseq (Anders and Huber, 2010), a state of the art software that models the null distribution of the count data with negative binomial distribution and evoluted variance. After normalization, we expected the miRNA counts to be highly correlated between samples corresponding to uninduced conditions (RU0) where the pathological proteins are not induced. This was indeed the case as illustrated in Figure 3 for samples issued from SCAs experiment. Therefore we could gain in statistical power by implementing, in the Deseq analysis, an additional set of reference data composed of independent samples corresponding to all the RU0 and other control conditions. This improved the accuracy of variance calculation and thus provides reduced *p*-values in the analysis. It also take into account the natural variation of miRNA expression in non-pathological flies of different genotypes, which should be distinguished to pathology related variations. However it should be emphasized that this procedure only improves the estimation of variation between samples of the same biological conditions and that, in all cases, comparisons and differential expression analysis are performed between flies of similar genotypes (induced or not induced) to eliminate genetic background variations. Results presented below have been obtained with this optimized method, although standard analysis (using one to one condition comparison) provides similar conclusions.

**Figure 4:**
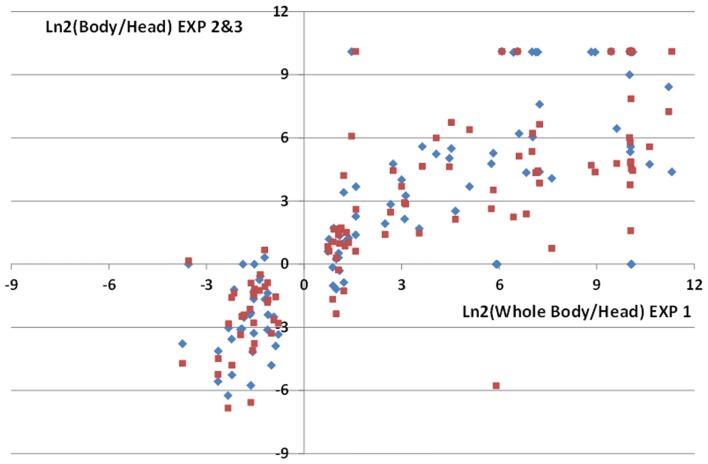
**Comparisons of miRNA enrichment or depletion in male fly heads**. Comparisons of ratios of normalized miRNAs read counts in fly bodies or heads were performed with 3 independent data sets: Exp1 (this study), Exp2 (Chung et al., 2008), Exp3 (Berezikov et al., 2011). We identified 106 statistically differentially expressed miRNAs in our analysis (Exp1 dataset). High correlations between Exp1 and Exp2 (diamonds) or Exp3 (squares) ratios for these miRNAs are observed. Notice that since decapitated flies were used to generate body extracts in Exp2 and 3, this results in lower Nbody/Nhead ratios for head enriched miRNAs compared to Exp1.

To validate our method of analysis we took advantage of the differences of miRNA expression observed in the different body parts. We performed deep-seq analysis on whole body of age matched flies, combined this data with our head control samples and used our pipeline to identify differentially expressed genes in the head. The raw and normalized counts of the miRNAs species are provided in Table S2 Supplementary Material. With our data, we identified 106 statistically significantly (*p* < 0.01) differentially expressed miRNAs, shared between 33 head enriched miRNAs (with ratios 0.08 < WholeBody/Head < 0.58) and 73 head depleted miRNAs (with ratios 1.66 < WholeBody/Head < infinite). Since similar datasets have been generated in two independent studies (Chung et al., 2008; Berezikov et al., 2011; accession numbers GSM286601, GSM286602, GSM322543, GSM399107) for the modENCODE project, we could compare our WholeBody/Head ratios with these two datasets. We observed a striking correlation between these three independent experiments with 96 out of our 106 differentially expressed miRNAs (91%) being confirmed by the modENCODE data (Figure 4 and Table S2 Supplementary Material). This suggests that our procedure may distinguish differentially expressed miRNAs with high reliability.

The full lists of raw and normalized counts of the 480 *Drosophila* miRNAs species are provided in Table S3 Supplementary Material for the different conditions corresponding to our neurodegenerative models.

### miRNA profiling in SCAs models

Transgenic lines used for SCA1 and SCA7 models (UAS-Atxn1-15Q, UAS-Atxn1-82Q, UAS-Atxn7-10Q, UAS-Atxn7-102Q) have been described and characterized earlier (Fernandez-Funez et al., 2000; Latouche et al., 2007). For SCA3 models we generated two new full length Atxn3 transgenic lines derived from constructs described in (Mueller et al., 2009), UAS-Atxn3-15Q and UAS-Atxn3-70Q. We checked that, similarly to expanded truncated form of Atxn3, expression of the pathological form in adult neurons of elavGal4^GS^/+; UAS-Atxn3-70Q flies reduced their lifespan (Figure 1B). These flies present also a progressive decline in climbing performance with a climbing index of 50% of control flies at 23 days and 14% at 30 days. In the same way, expression of the pathological form of ATXN1 in adult neurons of elavGal4^GS^/+; UAS-Atxn1-82Q flies reduced their lifespan (Figure 1C).

Importantly, all these SCAs related proteins have been implicated in transcription regulation. Atxn1 and Atxn7 are present in transcription complexes (Helmlinger et al., 2006; Lam et al., 2006) and Atxn3 binds to chromatin and may regulate gene expression by interacting with histone acetylases (Evert et al., 2006). Therefore these proteins are good candidates for a direct regulation of miRNA expression. We profiled miRNAs in two independent samples of total RNA extracted for heads of flies expressing the expanded Atxn proteins and 1 sample for uninduced control flies or flies expressing the normal allele, and performed multiple comparisons of expression with the Deseq software in standard mode. miRNAs presenting fold change greater than 1.5 between expanded protein expressing flies (RU200) and control flies (RU0) are given in Table S3 Supplementary Material. We noticed that many of these selected miRNAs present low expression values or, in some cases, high variability of expression between replicated samples or between the set of control samples. Consequently, in any case, we found no statistically significant difference (*p* < 0.01) in miRNA expression. This suggests that toxicity in the SCAs model in *Drosophila* is not related to misexpression of miRNAs mediated by the expanded Atxn proteins in the early stages of the disease. However, when we looked more closely to the miRNAs from Table 1 corresponding to conserved miRNA families, we noticed a trend to overexpression of mir-33 and mir-92a in all ataxia models (Table 2), although the statistical significance is below threshold in independent analysis. Closer analysis of these miRNAs at different time points coupled with functional analysis are under progress.

**Table 2:** **Fold changes observed after induction of the pathological proteins or treatments for miRNAs implicated in neurodegenerative diseases belonging to conserved miRNA families with *Drosophila* members**.

dme-miR	elavGS.M.200_ vs_elavGS.M.0	elavGS.F.200_ vs_elavGS.F.0	ATXN1.82Q.200_ vs_ATXN1.82Q.0	ATXN3.70Q.200_ vs_ATXN3.70Q.0	ATXN7.102Q.200_ vs_ATXN7.102Q.0	FUS.200_ vs_FUS.0	TDP43.WT.200_ vs_TDP43.WT.0	tau.V337F.200_ vs_tau.V337F.0	par.10_ vs_par.0	syn.A30P_ vs_elav	syn.WT_ vs_elav
	Fold change	Fold change	Fold change	Fold change	Fold change	Fold change	Fold change	Fold change	Fold change	Fold change	Fold change
dme-let-7_5p	0.96	0.94	1.14	1.03	1.15	0.77	0.76	0.95	0.78	0.75	0.84
dme-mir-1_3p	0.34	1.20	0.99	0.59	0.76	0.63	0.69	0.70	2.52	0.50	1.01
dme-mir-100_5p	1.23	1.02	1.50	1.61	1.44	1.23	1.18	1.20	1.40	1.10	0.80
dme-mir-125_5p	0.49	0.90	0.85	0.98	1.31	1.10	0.88	0.96	0.98	0.68	0.52
dme-mir-133_3p	1.50	0.92	1.02	1.16	1.31	0.75	0.72	0.61	0.91	0.96	0.77
dme-mir-137_3p	1.28	0.93	1.07	1.07	0.93	1.32	1.02	1.01	0.67	1.29	0.83
dme-mir-184_3p	0.40	1.13	0.83	0.77	0.70	0.75	0.57	0.57	1.45	1.36	0.97
dme-mir-193_5p	0.73	1.09	0.74	0.86	0.86	0.96	0.90	0.94	1.47	1.12	0.79
dme-mir-210_3p	0.59	0.96	0.79	0.81	0.75	0.87	0.69	0.85	0.98	1.22	0.87
dme-mir-285_3p	1.43	0.97	1.19	1.34	1.38	1.68	1.34	1.11	0.80	1.20	0.89
dme-mir-33_5p	1.22	1.04	1.89	1.54	1.79	1.45	1.09	1.37	0.53	1.55	0.91
dme-mir-34_5p	1.16	0.97	1.62	1.40	1.42	0.84	0.96	1.06	0.44	1.73	1.00
dme-mir-375_3p	0.46	0.91	1.32	0.90	0.59	1.16	1.28	0.77	1.11	1.02	0.88
dme-mir-92a_5p	0.91	0.74	1.74	2.00	1.90	1.68	1.90	1.78	1.89	0.93	1.19
dme-mir-92b_3p	1.63	1.18	1.47	0.90	0.80	1.33	1.06	0.92	1.25	0.79	1.16

*Variations greater than 1.5 are highlighted. In all cases, after Benjamini–Hochberg corrections for multiple testing, the DeSeq analysis *p*-value is 1. See Tables S2 and S3 in Supplementary Material for complete data*.

Recently a study in HD models identified 21 nt small CAG repeats RNAs (sCAG) as potential mediators of expanded Htt toxicity (Bañez-Coronel et al., 2012). Thus, in addition from miRNAs profiling, we searched for such species in our *in vivo* polyQ disease models by mapping all the sequences obtained in one model against the transgene used in this model. In all SCA diseases models we were unable to identify an increase in sCAG (Table 3). Indeed, only two perfect match reads of 18 and 21 nucleotides are detected in the ATXN7-102Q sample, while three and two imperfect match reads are detected in the ATXN1-82Q and ATXN3-70Q samples respectively. Thus, HD may present a specific toxicity mechanism compared to SCAs diseases, although alternative hypothesis discussed in the last section may explain our result.

**Table 3:** **No siRNAs like sequences are generated from CAG repeats**.

Sample	Coordinate polyQ	Orientation	Position	Sequence	N mismatch or insertion	Size
READS MAPPING INTO TRANSGENES						
ATXN1-82Q	1236–1482	+	1274	GCATCAGCATCAGCAGCA	2	18
ATXN1-82Q	1237–1482	–	1324	AGCAGCAGCAGCAACAG	1	17
ATXN1-82Q	1238–1482	+	1347	CAGCAGCATCAGCAGCA	1	17
ATXN3-70Q	1374–1584	+	1517	GCAGCATCAGCAACA	2	15
ATXN3-70Q	1375–1584	+	1561	AGCAGCAGCCGGAGCAGCAGC	2	21
ATXN7-102Q	735–1041	+	779	GCAGCAGCAGCAGCAGCA	0	18
ATXN7-102Q	735–1041	+	809	GCAGCAGCAGCAGCAGCAGCA	0	21
ATXN7-102Q	735–1041	+	821	ACAGCAGCAGCAGCAGCA	1	18
ATXN7-102Q	735–1041	+	952	AGCAGCAGCATCAGCA	1	16
ATXN7-102Q	735–1041	+	976	AGCAGCAGCAGCGGCA	1	16
ATXN7-102Q	735–1041	+	988	AGCATCAGCAGCAGCA	1	16
ATXN7-102Q	735–1041	+	1015	AGCAACAGCAGCAGCA	1	16
ATXN7-102Q	735–1041	–	1028	GGAGCAGCAGCAGCCG	2	16

	ATXN1-82Q	ATXN3-70Q	ATXN7-102Q			

**N total reads**						
**N reads in transgene**	751	1472	1632	
**N reads in repeat**	3	2	8	
**N reads in repeat-PM**	0	0	2	
**Reads/kb in transgene**	107	199	225	
**Reads/kb in repeat**	12	10	26	

*The small RNA libraries from the three expanded Atxn protein samples were mapped with Bowtie to the sequence of the corresponding transgene, to identify potential sequences generated from the CAG repeats. All the sequence identified as mapping into the repeat region are depicted here. Notice that the sizes of these sequences do not match with the 21 nt size expected for an siRNA and that most of them present some mismatches, which casts some doubt on their origin. In addition, as shown in the bottom Tab, we did not observe an increase of small RNA production in the repeat region, but, in contrast, a decrease compared to the rest of the transgene sequences*.

### miRNA profiling in FTLD models

Most cases of FTLD are characterized by the abnormal accumulation of either the microtubule-associated protein Tau, the transactive response DNA-binding protein-43 (TDP-43) or the FUS protein. TDP-43 proteins have been shown to facilitate the post-transcriptional processing of a subset of miRNAs not only in the nucleus but also in the cytoplasm (Kawahara and Mieda-Sato, 2012). This sequential facilitation was achieved both by the direct binding of TDP-43 to the primary and precursor forms of the miRNAs and by a protein–protein interaction between TDP-43 and the nuclear Drosha and cytoplasmic Dicer complexes. FUS proteins have also been involved in the Drosha complex, but the role of these proteins in miRNA processing has not been elucidated so far (Gregory et al., 2004). Therefore TDP-43 and FUS are good candidates for a direct regulation of miRNA expression. Expression of wild-type form of TDP-43 or FUS proteins was achieved using transgenic *Drosophila* lines (UAS-TDP-43, UAS-FUS) previously described and characterized (Miguel et al., 2011, 2012). miRNA expression profiling was performed in two independent samples of total RNA extracted from heads of flies expressing (RU200) or not (RU0) TDP-43 or FUS proteins. As shown in Table S3 Supplementary Material, similarly to SCAs models, miRNAs presenting fold change greater than 1.5 between induced and uninduced flies presented low expression values and/or high variability of expression between replicated samples or between the set of control samples, leading to none statistically significant difference (*p* < 0.01) in miRNA expression.

Concerning Tau proteins, it has been shown that suppression of miRNA maturation enhances Tau-mediated cell death in flies, indicating a protective role of miRNA in Tau neurotoxicity (Bilen et al., 2006). Furthermore, genetic ablation of Dicer in mice results in disease-like changes in endogenous Tau phosphorylation and neurodegeneration (Hébert et al., 2010). However there is no direct indication of miRNA dysregulations in flies expressing Tau. Transgenic lines used for FTLD-Tau model (UAS-TauV337M) have been described and characterized previously (Wittmann et al., 2001). In human, the V337M mutation had originally been described in a family (the Seattle family A) presenting an FTLD. Due to the X chromosomal insertion of the transgene, this particular set of experiments was performed using *Drosophila* females instead of males. A new set of control samples, corresponding to uninduced UAS-TauV337M/+; elavGS/+ samples (RU0); and elavGS/+ samples (RU0 and RU200), was therefore designed. Again, profiling of miRNA expression in two independent samples showed that miRNAs presenting fold change greater than 1.5 between induced and uninduced flies presented low expression values and/or high variability of expression between replicated samples or between the set of control samples, leading to none statistically significant difference (*p* < 0.01) in miRNA expression. However, like in ataxia models, mir-92a present trend to overexpression in all FTD models (Table 2), although the statistical significance is below threshold in independent analysis.

### miRNA profiling in PD models

We analyzed potential miRNA misregulation in two conditions that mimic Parkinson’s disease in flies. First, we treated the flies with paraquat, a toxicant suspected to be a factor of neurodegeneration in PD (Tanner et al., 2011) that is frequently used in animals to model PD (Cannon and Greenamyre, 2010). Paraquat produces free radicals in cells, which leads to dopamine neuron dysfunction and degeneration in *Drosophila* (Chaudhuri et al., 2007). Flies were treated with 20 mM paraquat for one-day, a dose that we found induces death of about half of the flies. Second, we used a transgenic model originally described by (Feany and Bender, 2000), in which the PD-implicated human protein α-*synuclein* or its more pathogenic mutant form α-*synuclein-A30P* were expressed in all *Drosophila* neurons starting from early stages of development. In this α-*synuclein* model, pathological features that include locomotor impairments and dopamine neuron loss only arise after 3 weeks of adult life (Feany and Bender, 2000). We performed our study on 7 to 10-day-old flies to detect potential early effects of α-synuclein neuronal accumulation on miRNA transcription.

As shown in Table S3 Supplementary Material we did not identified any statistically significant change in all the conditions analyzed. For instance, in the paraquat experiment, although mean fold changes between paraquat treated flies and control flies greater than two were observed rather frequently, they usually resulted from fluctuations in one of the four samples not confirmed in the other replicate. Compared to SCAs and FTLD studies where 100% flies are alive at the time of RNA extraction, higher variations in read counts may have resulted from greater heterogeneity in flies submitted to paraquat treatment that were recovered at the 50% survival time.

## Discussion

In this study, we investigated in several *Drosophila* models of neurodegenerative diseases whether miRNA misexpression may be observed after expression of toxic proteins (SCAs, FTLD, genetic PD) or toxic treatment (sporadic PD). In contrast to other profiling studies in model organisms or in humans, we measured miRNA expression at an early stage of the pathological process (3 days after toxic protein induction or treatment), in an attempt to uncover early transcriptional dysregulations linked to the diseases. However, no statistical significant variations in miRNA expression were observed in any of the cases.

Many studies previously reported differences in some miRNA expression in worm and mice models of AD, HD, PD or in post-mortem patient brain samples. In the latter case, a potential drawback of miRNA profiling is the limited stability and relatively short half-life (1–3.5 h) of some miRNAs (especially those with AT-TA content). Indeed, brain samples of post-mortem intervals (PMI) greater than 4 h and as high as 27 h hours (Martí et al., 2010) have been used in some studies, which may lead to overstated conclusions. Noticeably, miRNAs found differentially expressed in some of these studies (Martí et al., 2010; Wang et al., 2011) present higher percentages of AT-TA content (11%) than the complete miRNA collection (9%). Therefore, as already suggested by Sethi and Lukiw (2009), much care should be brought to sample collections and only experiments using samples with PMI < 2 h should be considered for future analysis and comparisons with animal models.

This potential problem of miRNA stability does not usually exist for analysis of samples from animal models (including our study) where fast freezing of tissues is performed. Thus, in addition to the reliable human data from low PMI samples, the reports of differential expression of miRNAs in worm and mice models for AD, HD, and PD are at odds with our findings, since they suggest that miRNA dysregulation is a common feature of neurodegenerative diseases. However, a significant difference with our study focused on the earliest stages of the pathological processes, is that miRNA profiling has been usually performed in aged animals where the pathological features are already present.

Since most of the diseases analyzed in our study with *Drosophila* models (SCAs, FTLDs) have not been submitted to similar miRNA profiling in mouse models, we cannot exclude the lack of miRNA dysregulation in these pathologies. Alternatively, the lack of significant miRNA dysregulation in our study may reflect the progressivity of the diseases. In this view the earliest stages following expression of a pathological protein would not be associated to changes in miRNA transcriptome while, at subsequent stages, some miRNAs would be dysregulated. Secondary events such as inflammation processes could play an important role in these latter steps. In agreement to this scheme miRNA-146a, a cytokine responsive miRNA, is induced in late stages of AD mouse models but not in younger animals (Li et al., 2011). Importantly, in contrast to miRNA, mRNA misregulation may be observed at early stages in at least some of our models. We and others have already documented early transcriptome changes as early as 12 h of paraquat treatment (Zou et al., 2000; Girardot et al., 2004). In addition, after completion of our study, one of us used the same RNA samples to perform mRNA deep-seq analysis on the 3 FTP models. Between 69 and 327 mRNAs were misregulated on these models, Although the description of this work is outside the scope of this paper, we confirmed here the induction of 5 chaperones by qRT-PCR analysis in the TauV337M model (Table 4). This shows that mRNA misregulation can occur 72 h after the pathological protein induction in at least four models used in this study.

**Table 4:** **Examples of deep sequencing analysis of mRNA species and validation by RT-qPCR**.

Gene id	Count A	Count A′	Count B	Count B′	Mean A	Mean B	Deep-seq fold change	padj	RT-qPCR fold change*	RT-qPCR fold change^‡^
*DnaJ-1*	1923	1697	2211	2891	1799	2530	**1.41**	2.4E-05	**1.33**	**1.37**
*Hsp83*	14887	13143	17140	22448	13939	19636	**1.41**	1.9E-05	**1.22**	**1.26**
*Hsp68*	483	430	497	945	452	718	**1.59**	3.0E-08	**1.64**	**1.70**
*Hsp70Bc*	110	95	164	393	101	277	**2.73**	1.7E-31	**1.32**	**1.37**
*Hsp70Aa*	342	247	663	1301	292	966	**3.31**	9.1E-61	**1.50**	**1.57**

*A: ElavGal4^GS^/+ RU200 #1; A′:ElavGal4^GS^/+ RU200 #2; B:ElavGal4^GS^/TauV337M RU200 #1; B′:ElavGal4^GS^/TauV337M RU200 #2*.

*: normalizer gene *14.3.3* ε; ^‡^: normalizer gene *Cyp1*.

*The 2 RU-induced FTLD-Tau samples and the 2 RU-induced female controls were submitted to deep sequencing of the mRNA species and analyzed as described previously. As a result, we observed that the 3 days-induced expression of Tau in *Drosophila* adult neurons induced a statistically significant misregulation of several genes among them heat-shock proteins for which we performed a validation by RT-qPCR, confirming the overexpression of these genes in the early stages of the disease. Fold changes in the two types of quantification are indicated in bold*.

In terms of potential therapeutic strategies it is important to elucidate whether miRNAs are early or late players in the neurodegenerative pathological processes. Therefore subsequent profiling experiments at different time points in the various animal models available are required to address this issue. In the case of *Drosophila* models particular attention should be brought to the two members of conserved mir family, mir-33, and mir-92a, that show trend toward overexpression in some models, albeit not statistically significant at this early time point. Notice, however, that, in these kinetic studies, conducted at the latter stages of the diseases, the issue of changes in tissue composition must be taken into account to avoid misleading interpretations.

As mentioned earlier, TDP-43 and FUS have been described as Drosha-associated protein. Furthermore, TDP-43 has been implicated in the production of a subset of precursor miRNAs (pre-miRNAs; Kawahara and Mieda-Sato, 2012) and its knockdown in culture cells can affect selected microRNA levels (Buratti et al., 2010). Strikingly, in this study we reported that TDP-43 or FUS overexpression for 3 days in adult differentiated neurons do not result in statistically significant difference in miRNA expression, indicating that an increase of the steady-state level of TDP-43 or FUS proteins *in vivo* do not modify the activity of Drosha complex 72 h after protein induction.

In addition to the miRNA profiling, we also analyzed for three polyQ disease models the generation of small (CAG)n RNAs. Surprisingly, we did not identify such species in our models. This contrast with the recent identification of 21 nt CAG repeat RNAs (sCAG) in cellular and mouse HD models that have been proposed to be mediators of expanded Htt toxicity (Bañez-Coronel et al., 2012). Although the sCAG may be specific to HD and related to the existence of an antisense transcript generated from a weak promoter (Chung et al., 2011), it is noticeable that untranslated long CAG repeats are toxic in mouse (Hsu et al., 2011), worm (Wang et al., 2011), and fly (Li et al., 2008). Therefore generation of toxic sCAG may be a general phenomenon in these species, relevant to all polyQ diseases. However, our result demonstrates unambiguously that these sCAG species are either not generated in young flies or are quickly eliminated. We suspect that age related changes may disrupt cellular homeostasis and lead to the progressive appearance of these toxic species. *In vivo* kinetics experiments are scheduled to check this hypothesis.

## Final Remark

The role of miRNAs in neurodegenerative diseases is still elusive in spite of an increasing number of reports. Importantly, most of these diseases are progressive and develop at old age. As our data suggest that miRNAs and sCAG may not be significantly involved in the early stages of these diseases, it urges for new longitudinal studies and genetic manipulations on animal models to better understand whether a progressive disruption of the production of these small RNA species may be truly relevant to neurodegenerative diseases.

## Conflict of Interest Statement

The authors declare that the research was conducted in the absence of any commercial or financial relationships that could be construed as a potential conflict of interest.

## Supplementary Material

The Supplementary Material for this article can be found online at http://www.frontiersin.org/Non-Coding_RNA/10.3389/fgene.2012.00226/abstract

Supplementary Table S1 **Distribution and percentage in the different RNA classes of the read counts obtained with the samples used in this study, as described in the Section “Material and Methods”**.

Supplementary Table S2 **Analysis of miRNAs expression in fly heads in independent experiments**. Deep sequencing was performed on age matched whole male flies with the same experimental methods than with fly heads. Raw data for body and head samples are provided in the first sheet. Normalized data including this experiment and data from (Chung et al., 2008; Exp2), and (Berezikov et al., 2011; Exp3) processed with the same analytical pipeline are given in the second sheet. A comparison of the fold changes between miRNA levels in bodies and heads in the three experiments is provided in the third sheet. The 106 miRNAs identified as statistically differentially expressed in our study are highlighted.

Supplementary Table S3 **Number of read counts without normalization (“raw data” sheet) or normalized (“norm data” sheet) for all the miRNA species identified in the samples of this study**. MirBase r18 was used for read assignment as indicated in the Section “Material and Methods”.

Supplementary Table S4 **Results of statistical analysis with Deseq**. For each of the comparisons analyzed for SCAs, FTLD, and PD models, the miRNAs with fold changes greater than two or lower than 0.5 are provided with the probability value for significant change calculated before (*p*-value) or after (padj) adjustement for multiple testing using the Benjamini–Hochberg (BH) method. On each line the minimum (Min), maximum (Max), medium (Mean), and standard deviation (SD) of the control samples used in Deseq variance analysis are given, in addition to the absolute values of the samples.
